# Methamphetamine treatment outcomes among gay men attending a LGBTI-specific treatment service in Sydney, Australia

**DOI:** 10.1371/journal.pone.0172560

**Published:** 2017-02-16

**Authors:** Toby Lea, Johann Kolstee, Sarah Lambert, Ross Ness, Siobhan Hannan, Martin Holt

**Affiliations:** 1 Centre for Social Research in Health, UNSW Australia, Sydney, New South Wales, Australia; 2 ACON, Sydney, New South Wales, Australia; University of Nebraska Medical Center, UNITED STATES

## Abstract

Gay and bisexual men (GBM) report higher rates of methamphetamine use compared to heterosexual men, and thus have a heightened risk of developing problems from their use. We examined treatment outcomes among GBM clients receiving outpatient counseling at a lesbian, gay, bisexual, transgender and intersex (LGBTI)-specific, harm reduction treatment service in Sydney, Australia. GBM receiving treatment for methamphetamine use from ACON’s Substance Support Service between 2012–15 (n = 101) were interviewed at treatment commencement, and after 4 sessions (n = 60; follow-up 1) and 8 sessions (n = 32; follow-up 2). At each interview, clients completed measures of methamphetamine use and dependence, other substance use, injecting risk practices, psychological distress and quality of life. The median age of participants was 41 years and 56.4% identified as HIV-positive. Participants attended a median of 5 sessions and attended treatment for a median of 112 days. There was a significant reduction in the median days of methamphetamine use in the previous 4 weeks between baseline (4 days), follow-up 1 (2 days) and follow-up 2 (2 days; p = .001). There was a significant reduction in the proportion of participants reporting methamphetamine dependence between baseline (92.1%), follow-up 1 (78.3%) and follow-up 2 (71.9%, p < .001). There were also significant reductions in psychological distress (p < .001), and significant improvements in quality of life (p < .001). Clients showed reductions in methamphetamine use and improved psychosocial functioning over time, demonstrating the potential effectiveness of a LGBTI-specific treatment service.

## Introduction

Approximately 2% of the Australian population report having used methamphetamine in the previous 12 months, a figure that has remained relatively stable over time [1]. However, there has been a recent trend towards the use of the more potent crystalline form of the drug (‘crystal methamphetamine’, hereafter ‘crystal meth’) over the less potent powder form (‘speed’) [1]. Among gay and bisexual men (GBM), the prevalence of any methamphetamine use in the previous 12 months is significantly higher than among heterosexual men in national, representative surveys (9.7% vs. 2.5%; adjusted odds ratio = 2.9) [2]. Community-based surveys of GBM in Australia have also reported high rates of methamphetamine use, with particularly high rates of crystal meth use reported among HIV-positive men (27.4% in the previous 6 months vs. 9.9% among HIV-negative and untested men) [3].

GBM often use crystal meth in sexual contexts, known colloquially as “chemsex” or “party and play”, and polydrug use with amyl nitrite, erectile dysfunction medications (e.g., Viagra) and gamma-hydroxybutyrate (GHB) is common [4–8]. Crystal meth plays an important role in forging social and sexual connections among some gay men, and is valued for its enhancement of sexual confidence, endurance and intimacy. However, using crystal meth in sexual contexts can increase the likelihood of engaging in high-risk sexual and drug use practices associated with HIV and hepatitis C virus (HCV) transmission, such as condomless anal intercourse with casual partners, multiple sex partners, group sex and injecting drug use [7, 9–14]. In a recent Australian study of GBM who injected drugs, 86% reported injecting crystal meth in sexual contexts and 41% reported sharing injecting equipment in the previous six months [15].

Compared to other methamphetamine forms, using crystal meth is associated with a higher likelihood of progressing to regular and heavier use, commencing injecting, and developing dependence [16]. In recent years, the average purity of crystal meth in Australia has dramatically increased (from 21% to 64% during 2009–13 based on seizures data), and while the cost per gram has also increased, the purity-adjusted cost has declined [17]. It has been suggested that the shift towards crystal meth use and changes in purity and cost have led to an increase in methamphetamine-related harms and burden on health services. The proportion of alcohol and other drug treatment episodes in Australia where methamphetamine was the principal drug of concern increased from 7% to 17% between 2009 and 2014 [18], and methamphetamine-related hospital admissions have substantially increased [19].

At present, treatment options for methamphetamine are limited. While a number of pharmacotherapies have been investigated in clinical trials, there are currently no approved medications for methamphetamine withdrawal or dependence [20, 21]. Psychosocial interventions, and cognitive behavior therapy (CBT) in particular, are currently regarded as the most effective treatments for methamphetamine users, and have been associated with the reduction or cessation of methamphetamine use and improved psychosocial functioning [22, 23]. As few as two sessions of CBT have been associated with improved treatment outcomes in clinical trials [24, 25].

People experiencing problems with methamphetamine use are often difficult to engage and retain in treatment due to perceptions that services do not offer methamphetamine-specific treatment, as well as high rates of relapse, polydrug use and mental health problems [26–28]. GBM may experience additional barriers to treatment, including concerns about discrimination from service providers and perceptions that mainstream services will have inadequate knowledge of the historical and current contexts of substance use and other issues among GBM [29–32]. GBM often report higher rates of substance dependence and psychological distress at treatment entry, and poorer treatment outcomes, compared to heterosexual men [33–36]. Some studies have reported better treatment outcomes in programs that have been culturally tailored to the needs of GBM, and many GBM report a preference for these services [31, 32, 37–39].

ACON, the largest community-based organization in Australia focused on the health and wellbeing of lesbian, gay, bisexual, transgender and intersex (LGBTI) people, provides an outpatient counseling service for LGBTI people seeking support to reduce or cease their use of alcohol and other drugs. The aim of the current study was to examine treatment outcomes among GBM seeking treatment for methamphetamine use from ACON’s Substance Support Service. We examined whether receiving treatment was associated with reduced methamphetamine use and related harms, and improved psychosocial wellbeing.

## Materials and methods

### Service description

ACON’s Substance Support Service is one of few LGBTI-specific alcohol and other drug treatment services in Australia, and is located in Sydney. An LGBTI-specific service is one that is specifically tailored to LGBTI people, where staff are often LGBTI-identifying, and provides culturally sensitive care that acknowledges the historical and cultural contexts of substance use among LGBTI people [40, 41]. The service recognizes the historical and current barriers to health and wellbeing experienced by LGBTI communities, as well as the potential challenges in finding an inclusive service that provides culturally appropriate care. The service is offered by a community organization that has a 30-year track record of working with and for LGBTI people.

ACON’s Substance Support Service provides free, short-term, outpatient, individual counseling for LGBTI people seeking assistance with their substance use. Treatment includes a structured intake interview (via telephone or in person with an intake officer or clinician), standard clinical assessment, and psychosocial intervention (typically up to 12 sessions). As this is a client-centred service, clinicians draw on range of therapeutic modalities (primarily acceptance and commitment therapy [42], as well as CBT and motivational interviewing) to tailor treatment to the needs of clients. Treatment is delivered within a harm reduction framework, and follows Australian clinical guidelines for methamphetamine treatment [23, 43]. The service recognizes that each client will have different treatment goals, which may include abstinence, reduction of use, or managing use.

During the study period, the service was staffed by two counselors, who have postgraduate qualifications in social work and counseling, respectively, and many years of experience working with LGBTI clients and people who use drugs. Both counselors have completed specialist training in evidence-based approaches to methamphetamine treatment.

### Participants

Eligible participants were men who: identified as gay or bisexual; were aged at least 18 years of age; and, were seeking treatment for methamphetamine use at the service between January 2012 and February 2015. There was no requirement that clients meet criteria for methamphetamine dependence in order to access the service or to be included in the present analysis, but methamphetamine had to be listed as the principal drug of concern in their client records. English language proficiency was a requirement for treatment due to limited resources to provide translation services. Only data from clients who had provided informed consent for their records to be analyzed and reported on in a non-identifiable format for research or evaluation purposes were included in this analysis. There were no other exclusion criteria.

There were an insufficient number of clients who presented with substances other than methamphetamine as their principal drug of concern, or who identified as lesbian, bisexual women, transgender or intersex, so the present study focused exclusively on GBM clients seeking treatment for methamphetamine use. Future publications may report on treatment outcomes for other clients and substances.

### Procedure

The Centre for Social Research in Health, UNSW Australia was approached by ACON to conduct a secondary analysis of treatment outcomes from the Substance Support Service. The study was approved by UNSW’s Human Research Ethics Committee and ACON’s Research Ethics Review Committee. Routinely collected quantitative data were collated by ACON and provided in a non-identifiable format to the Centre for Social Research in Health.

Participants self-completed questionnaires at treatment entry (baseline), and after every fourth counseling session. In this paper, we report data from interviews conducted at baseline (n = 101), after four sessions (follow-up 1; n = 60) and after eight sessions (follow-up 2; n = 32).

### Measures

At the baseline assessment, data were collected on demographic characteristics, methamphetamine and other substance use, methamphetamine dependence, primary route of methamphetamine administration, injecting drug use and risk of blood-borne virus transmission, and psychosocial functioning. At each follow-up interview, data were collected on methamphetamine and other substance use, methamphetamine dependence, injecting drug use and risk of blood-borne virus transmission, and psychosocial functioning.

#### Methamphetamine dependence

The Severity of Dependence Scale (SDS) measured methamphetamine dependence [44]. Summary scores on the SDS range from 0–15, with scores greater than 4 indicating methamphetamine dependence [45].

#### Psychological distress

The Kessler Psychological Distress Scale (K10) measured psychological distress in the previous 4 weeks [46]. Summary scores range from 10–50, with higher scores indicating higher levels of recent psychological distress. Using guidelines based on current Australian normative data, scores of 10–15 indicate low distress, 16–21 moderate distress, 22–29 high distress and 30–50 very high distress [47].

#### Quality of life

The 8-item European Health Interview Survey-Quality of Life (EUROHIS-QOL) index measured psychological, physical, social and environmental quality of life [48]. This is a short-form version of the World Health Organization Quality of Life Instrument—Abbreviated Version. Summary scores range from 5–40, with higher scores indicating better quality of life.

### Statistical analyses

Primary outcome measures were days of methamphetamine use in the previous 4 weeks and methamphetamine dependence at follow-up. Secondary outcome measures were other substance use in the previous 4 weeks, injecting drug use and risk practices, psychological distress and quality of life. Continuous and ordinal variables were examined for skewness and kurtosis, and none of these variables were normally distributed. For analyses involving these variables, medians and interquartile ranges were reported. Changes in methamphetamine use, other substance use, injecting drug use practices and psychosocial functioning between treatment commencement and 4 and 8 counseling sessions were analyzed using generalized estimating equation (GEE) models.

Baseline demographic, substance use, psychosocial functioning and treatment-related variables were compared between participants who did and did not reduce their days of methamphetamine use in the previous 4 weeks by at least 50% at follow-up 1. This analysis followed McKetin and colleagues’ analysis of treatment outcomes among a predominantly heterosexual sample of people receiving treatment for methamphetamine use [49], using Mann-Whitney U tests for continuous and ordinal variables, Pearson’s chi-square tests for categorical variables, and Fisher’s exact tests for categorical variables with expected cell counts of < 5. Two-tailed statistical significance of p < .05 was used for all comparisons. Analyses were conducted using Stata Version 13.1 (StataCorp, College Station, TX, USA).

## Results

### Sample characteristics

One hundred and one clients were included in this analysis. The median age of the sample was 41 years (interquartile range [IQR] 31–44; range 22–61). All but one participant identified as gay, while the remaining participant identified as bisexual. Most participants were born in Australia (69.3%), and 3 participants identified as Aboriginal and/or Torres Strait Islander. Half of participants were in full-time employment (52.5%), 6.9% were in part-time employment, 27.7% were receiving social welfare payments and 5.0% had no income. According to self-report, 56.4% of participants were HIV-positive, 36.6% were HIV-negative, and 6.9% were untested or of unknown HIV status. HIV-positive participants were significantly older than HIV-negative and untested/unknown status participants (median 43 vs. 36 years; z = -3.55, p < .001). There were no other differences in baseline characteristics according to HIV status.

### Treatment commencement

#### Methamphetamine and other substance use

The majority of participants had used methamphetamine in the 4 weeks prior to commencing treatment (82.2%), and had used methamphetamine on a median of 4 days over this period (IQR 2–9). The median SDS score at treatment entry was 8 (IQR 6–11) and 92.1% of participants had SDS scores indicating methamphetamine dependence.

The most common route of methamphetamine administration was injecting (58.4%), followed by smoking (32.7%). Two participants reported snorting and one participant reported ingesting as their main route of administration (6 participants did not provide this information). Seventy-nine percent of participants reported a history of injecting drug use, and 64.4% reported injecting in the previous 3 months. Among participants who had injected in the previous 3 months (n = 65), 21.5% reported receptive sharing of a needle or syringe (i.e., using equipment after someone else had used it), and 43.1% had shared other injecting equipment in the previous 3 months.

Seventy percent of participants had consumed alcohol in the 4 weeks prior to commencing treatment, 23.8% had smoked tobacco daily and 24.8% had used cannabis. Twenty-eight percent of participants had used illicit drugs other than cannabis (see Table 1).

**Table 1:** Methamphetamine use, other substance use, and psychosocial outcomes after four counseling sessions (follow-up 1) and eight counseling sessions (follow-up 2) compared to treatment entry (baseline).

	Baseline (n = 101)	Follow-up 1 (n = 60)	Follow-up 2 (n = 32)	OR (95% CI)	*p* value
Methamphetamine use in past 4 weeks					
Any use (%)	82.2	56.7	62.5	0.87 (0.81–0.94)	< .001
Days used (median)	4	2	2	0.23 (0.10–0.54)	.001
SDS score (median)	8	6	5	0.20 (0.12–0.34)	< .001
SDS dependence (%)	92.1	78.3	71.9	0.90 (0.85–0.95)	< .001
Other substance use in past 4 weeks (%)					
Any alcohol	70.3	80.0	78.1	1.02 (0.97–1.08)	.48
Medium- or high-risk alcohol^a^	15.8	20.0	18.8	0.94 (0.90–0.99)	.01
Daily tobacco	23.8	13.3	25.0	0.98 (0.94–1.03)	.50
Cannabis	24.8	21.7	18.8	0.96 (0.90–1.02)	.15
Benzodiazepines	18.8	31.7	28.1	1.03 (0.97–1.09)	.39
Cocaine	7.9	5.0	12.5	1.01 (0.96–1.06)	.70
Other illicit drugs	24.8	25.0	31.3	1.00 (0.93–1.06)	.89
Injecting drug use in past 3 months (%)					
Injected any drug	64.4	61.7	62.5	0.97 (0.92–1.02)	.25
Receptive sharing of needle / syringe	13.9	10.0	18.8	0.98 (0.95–1.00)	.051
Shared other injecting equipment	27.7	16.7	21.9	0.92 (0.87–0.98)	.007
Psychosocial functioning					
K10 score (median)	26	21.5	16.5	0.04 (0.01–0.12)	< .001
High / very high K10 score^b^ (%)	72.3	50.0	31.3	0.83 (0.77–0.89)	< .001
EUROHIS-QOL-8 score (median)	21	25	24	9.06 (3.87–21.23)	< .001

^a^Medium risk: 15–28 standard drinks per week; high risk: > 28 standard drinks per week.

^b^High K10 score: 22–29; very high K10 score: 30–50.

CI, confidence interval; EUROHIS-QOL-8, World Health Organization Quality of Life Instrument-Abbreviated Version, Short-Form; K10, Kessler Psychological Distress Scale; OR, odds ratio; SDS, Severity of Dependence Scale.

#### Psychosocial functioning

The median K10 score was 26 (IQR 20–32) at treatment entry. Thirty-seven percent of respondents had a K10 score indicative of high psychological distress in the previous 4 weeks and 35.6% had a score indicative of very high psychological distress. The median EUROHIS-QOL-8 score was 21 (IQR 17–27) at treatment entry.

### Treatment outcomes

Thirty-one percent of participants had previously received alcohol and/or other drug treatment at any service, and 25.7% had previously received treatment at ACON. Most participants (67.3%) had referred themselves into treatment. Participants attended a median of five counseling sessions (IQR 2–9) and attended treatment for a median of 112 days (IQR 42–169). A median of 47 days (IQR 35–73) elapsed between baseline and follow-up 1, and 119 days (IQR 101–141) between baseline and follow-up 2.

#### Methamphetamine and other substance use

There was a significant reduction in any methamphetamine use, days of methamphetamine use and methamphetamine dependence at follow-up compared to baseline (see Table 1). Among participants who reported methamphetamine use in the 4 weeks prior to treatment commencement, abstinence from methamphetamine was reported by 35.3% (n = 18) of participants at follow-up 1, and by 33.3% (n = 9) of participants at follow-up 2.

There was also a significant reduction in the proportion of participants who reported sharing ancillary injecting equipment at follow-up compared to treatment commencement. There was a reduction in the proportion of participants reporting medium- or high-risk alcohol use in the previous 4 weeks at follow-up compared to treatment commencement (see Table 1).

#### Psychosocial functioning

Among participants at both follow-up interviews, there was a significant reduction in K10 scores in the previous 4 weeks compared to treatment commencement, and a significant reduction in the proportion of participants who reported high or very high levels of psychological distress. There was also a significant improvement in overall quality of life among participants at follow-up compared to treatment commencement (see Table 1).

### Participants who reduced methamphetamine use

Participants who reduced their days of methamphetamine use by 50% at follow-up 1 compared to baseline reported significantly lower median quality of life scores at treatment commencement compared to other participants (see Table 2). There were no other variables that were significantly associated with reduced methamphetamine use at follow-up.

**Table 2:** Comparison of baseline characteristics of participants according to whether they had reduced their methamphetamine use after four counseling sessions (follow-up 1).

	Reduced methamphetamine use by at least 50%^a^		
	No (n = 21)	Yes (n = 30)	*p* value
Demographics			
Age (median)	38	41	.13
Born in Australia (%)	71.4	70.0	.91
Paid employment (%)	76.2	70.0	.63
HIV-positive (%)	57.1	56.7	.97
Treatment			
First treatment episode (%)	14.3	26.7	.29
Number of sessions (median)	7	8	.80
Days in treatment (median)	143	141	.57
Methamphetamine use in past 4 weeks			
Days used (median)	4	7	.11
SDS score (median)	8	9	.09
SDS dependence (%)	95.2	96.7	.80
Other substance use in past 4 weeks (%)			
Medium- or high-risk alcohol use^b^	19.0	33.3	.26
Daily tobacco use	23.8	13.3	.33
Cannabis	38.1	20.0	.15
Benzodiazepines	38.1	23.3	.26
Cocaine	14.3	6.7	.37
Other illicit drugs	38.1	30.0	.55
Injecting drug use in past 3 months (%)			
Injected any drug	76.2	63.3	.33
Receptive sharing of needle / syringe	19.0	16.7	.83
Shared other injecting equipment	33.3	33.3	1.0
Psychosocial functioning			
K10 score (median)	23	26	.22
High / very high K10 score^c^ (%)	61.9	76.7	.26
EUROHIS-QOL-8 score (median)	25	20	.03

^a^Participants who were abstinent at treatment entry were excluded from these analyses.

^b^Medium risk: 15–28 standard drinks per week; high risk: > 28 standard drinks per week.

^c^High K10 score: 22–29; very high K10 score: 30–50. EUROHIS-QOL-8, World Health Organization Quality of Life Instrument-Abbreviated Version, Short-Form; K10, Kessler Psychological Distress Scale; SDS, Severity of Dependence Scale.

### Characteristics of clients completing follow-up interviews

Compared to participants who did not complete a follow-up interview, participants who completed a follow-up interview were significantly more likely at treatment commencement to be in paid employment (68.3% vs. 43.9%; p = .01) and to have consumed alcohol at medium- or high-risk levels in the previous 4 weeks (26.7% vs. 0.0%; p < .001).

## Discussion

In this sample of GBM receiving outpatient counseling for methamphetamine use, we found significant reductions in methamphetamine use and methamphetamine dependence, and significant improvements in psychosocial wellbeing, after four and eight treatment sessions. This is the first study to report on treatment outcomes among GBM attending a LGBTI-specific alcohol and other drug treatment service in Australia [50].

While this study provides externally valid evidence from an existing treatment service, there were some important methodological limitations. Firstly, there was no control group (either standard non-LGBTI-specific treatment, or a group receiving no treatment), so we cannot determine the extent that the positive outcomes were due to factors other than the treatment received, and the relative importance of attending a LGBTI-specific service. However, findings from a small number of randomized controlled trials in the US suggest that GBM experience more substantial declines in methamphetamine use in treatment programs tailored to GBM [36, 37]. Secondly, there was a high rate of attrition, which may have affected the treatment outcomes reported at each follow-up as data were not collected from participants who discontinued treatment before completing a follow-up interview. However, we found no differences at treatment entry between participants who did and did not complete a follow-up interview in terms of methamphetamine use, methamphetamine dependence and psychosocial wellbeing. Thirdly, loss to follow-up meant that there was limited statistical power to examine differences between participants who did and did not report significant reductions in methamphetamine use. This may help to explain why only one factor, quality of life at baseline, was associated with reduced methamphetamine use at follow-up. In other studies, more regular methamphetamine use, greater severity of dependence and injecting drug use have been associated with poorer treatment outcomes [28, 51]. Finally, data about sexual practices were not collected in the routine questionnaires. Given that methamphetamine is commonly used by GBM to enhance sexual experiences and can be associated with HIV risk practices and transmission [52], it is unfortunate that we were unable to determine whether sexual risk practices changed during the course of treatment. This study was conducted with limited resources, and improved survey measures as well as a qualitative component will be included in a full evaluation, subject to funding.

The harm reduction approach of ACON’s Substance Support Service supports both abstinence and reduction as treatment goals. While we reported significant reductions in methamphetamine use after 4 and 8 treatment sessions, more than half of participants reported some methamphetamine use at follow-up. This finding is similar to what has been reported in other studies of people receiving outpatient counseling for methamphetamine [24, 25, 49], including studies of GBM [36, 37, 53].

During the study period, the service was primarily attended by gay men, highlighting a need for better treatment engagement with bisexual men, lesbian and bisexual women, and trans and other sex and gender diverse people who are experiencing problems with alcohol and other drugs. The high proportions of clients who were gay and HIV-positive men could reflect that these men currently experience a greater burden of methamphetamine-related harms in Sydney, but also may be in part attributed to ACON’s history as a HIV organization. The high proportion of HIV-positive participants and participants reporting injecting as their primary route of methamphetamine administration is consistent with the high rates of crystal meth use and injecting drug use reported among HIV-positive gay men in community-based surveys in Australia [3]. Crystal meth use has been associated with a higher likelihood of engaging in high-risk sexual practices [14], and among HIV-positive men, the sexual transmission of hepatitis C [54], and poor adherence to HIV antiretroviral treatments [55]. Making methamphetamine treatment accessible and attractive to GBM experiencing problems from use is thus a priority for blood-borne virus prevention.

While most LGBTI people who use methamphetamine do not develop problems from use, there remains a need for services that are tailored specifically for LGBTI people, have detailed knowledge of LGBTI issues, and provide access to LGBTI clinicians [56]. While LGBTI-specific services fulfill an important role and are preferred by many LGBTI people [31, 33, 38], these services may not be accessible to people living outside of major cities. The few tailored services available to LGBTI people in Australia are at or near operating capacity [57, 58]. Ongoing funding is essential to ensure that these services are accessible to the people who need them.

To improve access to quality alcohol and other drug treatment services for GBM and other LGBTI people, there is a need for wider coverage of mainstream treatment services that are sensitive to the cultural contexts of substance use and other issues experienced by LGBTI people (known as LGBTI-sensitive services). Mainstream services will see LGBTI clients, so it is essential that the these services have the appropriate skills and training to provide culturally appropriate care to LGBTI clients. It is also critical that accurate sexuality and gender indicators are included in the data collection tools that mainstream services utilize; without their use LGBTI people will remain invisible in mainstream clinical data sets. While some community-based LGBTI organizations in Australia provide cultural competency training for mainstream service providers, additional resources are required to support mainstream services in working effectively with LGBTI people to ensure widespread access and availability of these services.
